# The Bactericidal Activity of Protein Extracts from *Loranthus europaeus* Berries: A Natural Resource of Bioactive Compounds

**DOI:** 10.3390/antibiotics9020047

**Published:** 2020-01-28

**Authors:** Rosa Luisa Ambrosio, Lorena Gratino, Sara Mirino, Ennio Cocca, Antonino Pollio, Aniello Anastasio, Gianna Palmieri, Marco Balestrieri, Angelo Genovese, Marta Gogliettino

**Affiliations:** 1 Department of Veterinary Medicine and Animal Production, University of Naples Federico II, 80137 Napoli, Italy; rosaluisa.ambrosio@unina.it (R.L.A.); anastasi@unina.it (A.A.); 2 Institute of Biosciences and BioResources, National Research Council (IBBR-CNR), 80131 Napoli, Italysara.mirino@gmail.com (S.M.); marco.balestrieri@ibbr.cnr.it (M.B.); marta.gogliettino@ibbr.cnr.it (M.G.); 3 Department of Biology, University of Naples Federico II, 80126 Napoli, Italy; anpollio@unina.it (A.P.); genovese@unina.it (A.G.)

**Keywords:** *Loranthus europaeus*, Protein extract, Antibacterial agents, Natural compounds, *Staphilococcus aureus*

## Abstract

*Loranthus europaeus* is a well-known and important medicinal plant, with a long history of traditional medicine use. Several studies showed that it contains many bioactive compounds with a wide range of pharmacological effects. In light of these past researches, *L. europaeus* were chosen to consider its potential antimicrobial action. To this aim, different protocols were performed to selectively extract protein compounds, from *L. europaeus* yellow fruits, and evaluate the antimicrobial activity against four phytopathogenic fungi (*Aspergillus niger*, *Alternaria* spp., *Penicillium* spp., *Botritis cinereus*) and a number of foodborne bacterial pathogens (*Listeria monocytogenes*, *Staphylococcus aureus* strains, *Salmonella* Typhimurium and *Escherichia coli*) by using serial dilutions and colony formation assays. Results evidenced no antifungal activity but a notable bactericidal efficiency of a crude protein extract against two foodborne pathogens, with minimum inhibitory concentration (MIC) and minimum bactericidal concentration (MBC) values between 0.2 and 0.5 mg/mL, being *S. aureus* strains the most susceptible bacteria. Moreover, a strong bactericidal activity against *S. aureus* M7 was observed by two partially purified protein fractions of about 600 and 60 kDa molecular mass in native conditions. Therefore, these plant protein extracts could be used as natural alternative preventives to control food poisoning diseases and preserve foodstuff avoiding health hazards of chemically antimicrobial applications.

## 1. Introduction

In chapter XVI, 95 of the *Naturalis Historiae*, Pliny the Elder described the harvesting of the mistletoe growing on the oaks by Gaul druids, a cult related to the medicinal and magic properties attributed to mistletoes [1]. Indeed, these plants were highly reputed as a remedy for many ills, and specifically effective *contra venena* [2], but the traditions about magic and curative properties of mistletoes are not confined to Central-Northern Europe. The anthropologist James G. Frazer in his land-marking “The Golden Bush” [3] presented a detailed survey of the symbolic role of mistletoes in the ancient Italic cult of Diana Nemorensis, and in the 1900s a growing evidence has been presented on their occurrence as sacred plants in folklore and mythology of several Indo-European cultures [4]. 

Mistletoe is a generic term encompassing all the obligate hemiparasitic species of Angiosperms, presently placed in five phylogenetically unrelated different families, within the order Santalales. Loranthaceae and Viscaceae are the families that include most of mistletoes worldwide diffused [5]. Archaeological findings support the use of *Viscum album* in the religious cults of druids in England, between the first and second century AD [6], but *V. album* is not the only mistletoe diffused in Europe. Indeed, magic and curative properties were attributed also to other mistletoe species *Loranthus europaeus*, the “true” golden bush, prevalently diffused in the Mediterranean territories of the Continent [7]. According to Liu et al. [8], the Loranthaceae originated in Australasia during Late Cretaceous, when these two Continents were connected or contiguous, and also the genus *Loranthus* has an Australasian origin, but dispersed also to North Asia and Europe. In the course of the last Century, the majority of *Loranthus* species has been transferred to different genera [9], and presently the genus is represented by about ten species [10]. European yellow mistletoe, *L. europaeus* Jacq., is the only species of the genus that migrated westward to Europe, and is presently diffused in Central Asia, Anatolia, South Russia, and South-Western Europe [11]. *L. europaeus* (European yellow mistletoe) is a small shrub with brown bark and deciduous leaves during the winter, with yellowish-green flowers. The fruit is a spheroidal golden-yellow berry, with a sticky liquid inside. As the other hemiparasitic Santalales, *L. europaeus* actively photosynthesizes, but gains from the host plants water, inorganic nutrients and also organic compounds, such as amino acids and sugars [12]. *L. europaeus* can establish a relation with different *Quercus* species, with *Q. pubescens* being by far the first choice host plant, although this mistletoe can also attack other trees such as chestnut [13]. *L. europaeus* is presently used to treat many numerous ailments in the folk medicine of different Asiatic and European Countries [14,15]. In several regions of Central Italy, the whole plant macerated in wine or grappa was used to cure atherosclerosis and hypertension [16], whereas in Calabria region (South Italy) leaves were also topically applied to cure wounds [17]. The therapeutic effects of *L. europaeus* have been attributed to the presence of a wide array of active substances: the mixture of flavonoids isolated from the plants has shown marked antioxidant properties [18,19], and a stimulatory effect on lymphocyte proliferation has been attributed to flavonoids and terpenoides isolated from the leaves [20]. Recently, an anti-leishmaniasis effect has been attributed to the presence a high concentrations of quercetin in the whole plant extract [14]. The antimicrobial and cytotoxic activities attributed to *L. europaeus* could be also due to the presence of the so-called defense peptides, more recently also known as plant defensins, largely occurring in different genera of parasitic members of Santalaceae and Loranthaceae [21]. Plant defensins have shown a specific activity not only against pathogenic fungi, but also to yeast models [22], and for this reason are considered a possible source of therapeutic compounds also against human fungal infections [23].

The insights gained from this work demonstrated, for the first time, the antimicrobial activity of a crude protein extract from the European medicinal plant *L. europaeus* against the Gram-positive bacteria *Staphylococcus aureus* and *Listeria monocytogenes*. The bactericidal effect of two partially purified protein compounds isolated from the yellow berries was also examined against the methicillin-resistant *S. aureus* M7, whose growth was completely inhibited already at a concentration of 0.01 mg·mL^−1^. It should finally be noted that this study started thanks to the correlation of the anthropological and biogeographic data made by the coauthor of the manuscript Angelo Genovese. 

## 2. Results

### 2.1. Samples Collection

**Figure 1:**
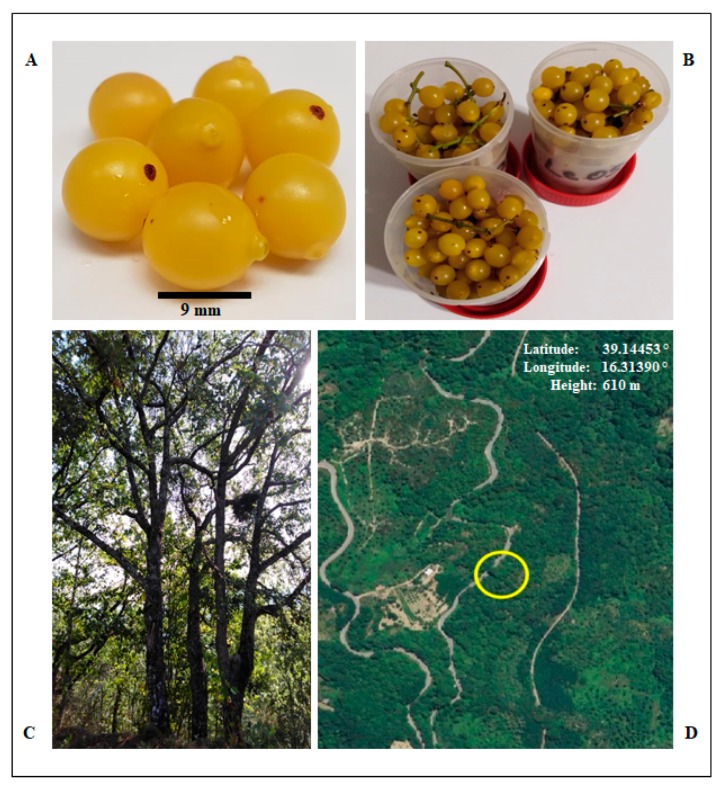
(**A,B**) Ripen berries of *L. europaeus*; (**C**) oak tree hosting *L. europaeus* twigs; (**D**) the sampling site (Carpanzano forest, Calabria, Italy).

In Italy, *Loranthus europaeus* is prevalently diffused in oak forests of Apennines, extending from Central to South Italy. For this study, the forest of Carpanzano (Calabria), located at an altitude of 610 m, was selected. Carpanzano is a continental territory of Calabria, far from the sea, with cold winters and higher precipitations during spring and fall. Visible tufts of *L. europaeus* were scattered on numerous *Q. pubescens* trees and samples were collected during winter, when mistletoe twigs are leafless, and fruits acquire a bright yellow color (Figure 1).

### 2.2. Preparation of Protein Extracts from *Loranthus europaeus* Berries and Plant Extracts Yield

With the aim to recover and isolate new plant protein agents with antimicrobial activity, that can possibly be used as natural preservatives in the food and pharmaceutical industries, an efficient protein extraction method from *Loranthus europaeus* berries was developed. Indeed, the extraction efficiency is strongly affected by several factors such as the starting plant material, the buffer composition and the method used as well as the presence of interfering substances [24]. It is worth noting that at present there are relatively few reports on the extraction protocols of antibacterial proteins from plant berries respect to those on the wide variety of smaller molecules, obtained usually through ethanol or methanol extraction [25].

**Figure 2:**
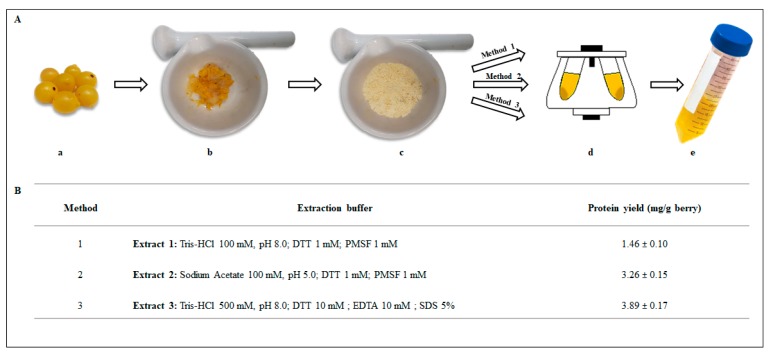
(**A**) Representative scheme of the different steps applied for the preparation of crude protein extracts from the yellows berries of *L. europaeus*. **a**: yellow berries; **b**: pitted berries; **c**: powdered berries in liquid nitrogen using a mortar and pestle; **d**: centrifugation of the mixture; e: crude protein extract. Finely ground powder of plant fruit was used as starting material in all three protocols. (**B**) Table reporting protein yields from berries of *L. europaeus* using three extraction protocols. Data are presented as means ± standard deviation (s.d.) of three different samples analyzed in triplicate.

In this study, three of simply, fast and common extraction protocols used for proteins were carried out for berries with some modifications [26,27,28,29], taking into account both the pH and the presence of strong anionic detergents such as SDS (Figure 2A). As far as the protein recovery is concerned, the quantitative comparison among the different extracts showed that the highest yield was obtained with method 3, followed by method 2 and method 1 that gave the lowest protein yield (Figure 2B). However, it is worth noting that the protein quantitation assay on extracts from protocol 3 was affected by the presence of SDS-containing buffer that persisted even after extensive dialysis of the sample, thus interfering with the protein yield results [30]. 

**Figure 3:**
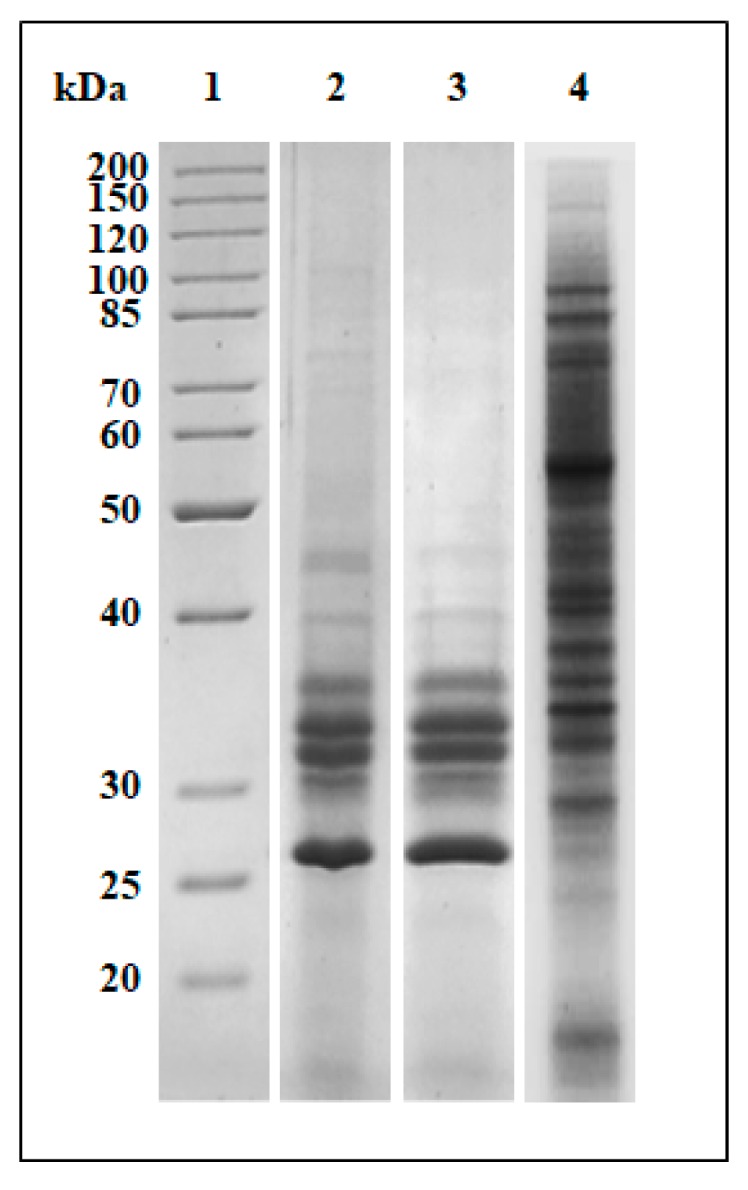
SDS-PAGE (10%) analysis of the total protein extracts from *L. europaeus* berries using the three methods. Lane 1: molecular weight markers (Thermo Scientific); crude protein extracts obtained by: method 1 (Lane 2); method 2 (Lane 3); and method 3 (Lane 4). Protein bands were detected by Coomassie blue staining. Equal amounts of proteins were loaded for each Lane. The gel is representative of three independent experiments on three different protein preparations.

The protein pattern of the three extracts was assessed by SDS-PAGE analysis and a representative Coomassie-stained gel is reported in Figure 3. The protein extracts 1 and 2 showed a similar electrophoretic profile in contrast to that associated with the extract 3, possibly resulting from the use of SDS in the extraction buffer, which is known to be extremely effective in the solubilization of membrane proteins [31].

### 2.3. Antifungal Activity

**Figure 4:**
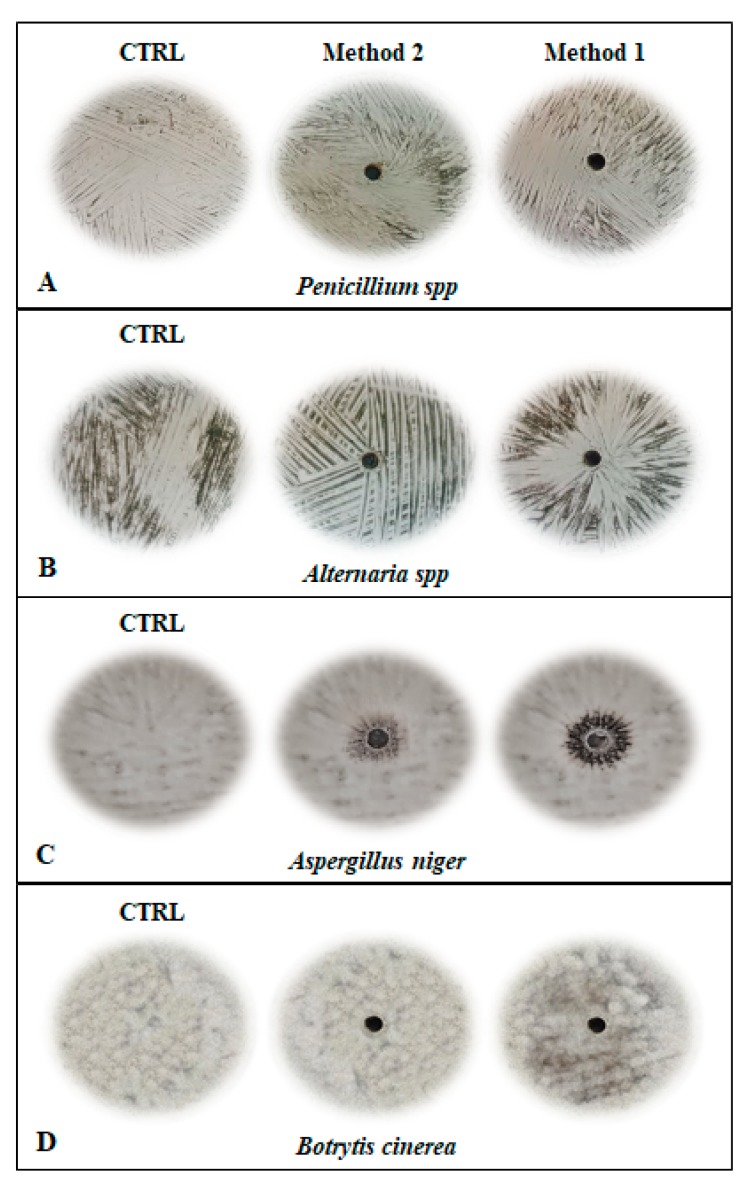
Antifungal activity assay of plant extract 1 (Method 1) and plant extract 2 (Method 2) against different phytopathogenic fungi: (**A**) *Penicillium* spp; (**B**) *Alternaria* spp; (**C**) *Aspergillus niger*; (**D**) *Botrytis cinerea*. CTRL: each tested fungus without treatment. The plates were incubated at 28 °C for 48 h. The pictures are representative of three independent experiments on three different protein preparations.

An initial in vitro screening was done to evaluate the antifungal activity of all the plant extracts against four of the most common phytopathogenic fungi. As depicted in Figure 4, none of the two extracts at pH 8.0 and 5.0 (Method 1 and 2) showed antifungal activity against any of the test microorganisms, even at the highest amount investigated. In addition, the two protein samples seemed to promote the sporulation of *Aspergillus niger* (colony diameter of 3.5 ± 0.3 cm and 2.0 ± 0.8 cm for extract 2 and extract 1, respectively after 48 h of incubation), possibly due to the presence in the plant extracts of some additional nutrients which could further stimulate the fungal growth (Figure 4C). As far as the SDS-extract (Method 3) is concerned, it is worth noting that even after extensive dialysis of the samples, a residual amount of detergent persisted in the protein mixtures, resulting in a strong interference with the antifungal and antibacterial activity assays, which require a complete removal of this detergent. For these reasons, the SDS-extracts were not further considered for our investigations. 

### 2.4. Antibacterial Activity

**Figure 5:**
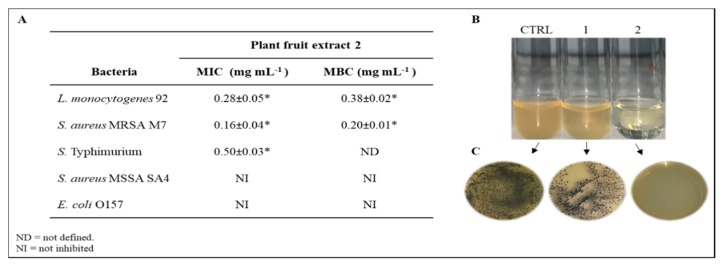
(**A**) Table of minimum inhibitory concentration (MIC) and minimum bactericidal concentration (MBC) values of plant fruit extract 2 against different foodborne pathogens. (**B**) Antimicrobial test in vitro of plant fruit extract 2 against *S. aureus* MRSA M7. **CTRL**: *S. aureus* MRSA M7 control; (**1**) protein extract 2 at 0.08 mg·mL^−1^ concentration; (**2**) protein extract 2 at 0.16 mg·mL^−1^concentration (MIC value). (**C**) MBC value (0.2 mg·mL^−1^) determined by the standard plate count. Data are presented as means ± standard deviation (s.d.) of three different samples analyzed in triplicate. * Significant difference (*p* < 0.05) between the treated and the control samples.

In order to explore the potential use of the protein samples as antimicrobial agents, the antibacterial activity of the extracts 1 and 2 was evaluated against a panel of bacteria, including 3 strains of Gram-positive *(L. monocytogenes*, *S. aureus* MSSA, and MRSA) and 2 strains of Gram-negative bacteria (*Salmonella* and *E. coli*), among those commonly associated with infectious diseases. Specifically, to compare the effect of the two extracts on the growth of the microorganisms under investigation, the MIC and MBC values were determined by using the serial dilution assay. It is known that sodium acetate can affect the bacterial growth [32,33], therefore preliminary experiments were performed in order to assess the effects of different concentrations of acetate on the growth of the foodborne pathogens, considering that the extract 2 was obtained using acetate as extractant. The obtained results evidenced a linear decrease of bacterial growth rate with the increase in acetate concentration starting from 60 mM (data not shown). For this reason, all the subsequent experiments with extract 2 were performed only after dialysis of the sample in order to have a final concentration of 50 mM acetate that did not interfere with the antimicrobial assays. Interestingly, the tested microorganisms revealed a different sensitivity to the two types of extracts. Overall, the results demonstrated that the extract 1 was less effective in suppressing the microbial growth of all pathogens tested, exhibiting MIC values 2-fold higher than those observed for the acetate-extract. It can be hypothesized that the variation in MIC values between the two plant-fruit samples arose from a diverse nature of the proteins extracted by using the acetate respect to the Tris buffer. Hence, the extract 1 was not considered for any further study based on its weak antibacterial activity. As far as plant fruit extract 2 is concerned (Figure 5A), it exhibited an efficient and significant antimicrobial activity against *L. monocytogenes, S. aureus* MRSA, and *S*. Typhimurium, with MIC values ranging from 0.16 to 0.50  mg·mL^−1^ , being *S. aureus* MRSA the most sensitive bacterial species. Indeed, the protein sample was found to be ineffective against *E. coli* and *S. aureus* MSSA SA4 even at the highest amount (0.50 mg·mL^−1^) assayed. To investigate further the antimicrobial effects of the extract 2, the MBC was evaluated revealing that it displayed a strong bactericidal activity against *L. monocytogenes* and S. aureus MRSA, with MBC values of 0.38 and 0.20 mg·mL^−1^, respectively. These results clearly indicated that this protein extract was bacteriostatic at concentrations lower than those required to explain bactericidal activity against *L. monocytogenes*, being MBC value higher than the corresponding MICs. Instead, the MBC determined against *S. aureus* MRSA was on a par with the corresponding MIC (both at about 0.2 mg·mL^−1^), thus demonstrating that the tested sample should be considered to have a strong bactericidal mode of action. On the other hand, *S.* Typhimurium needed protein concentrations higher than 1 mg mL^-1^ to be killed, indicating that the active substances were only bacteriostatic towards this strain. Therefore, according to the results obtained, the Gram-positive bacteria were more sensitive to the plant extract 2 than the Gram-negative microorganisms, presumably as consequence of the different bacterial membrane structures. Specifically, lipopolysaccharides layer and periplasmic space of Gram-negative bacteria could be the reasons of the relative resistance of this class of bacteria to the plant extract 2 treatment. However, this explanation represents a simplification as other mechanisms could play a role in this process. Interestingly, in relation to the antibacterial spectrum of the crude extract (Figure 5A), it is important to emphasize the strong growth inhibition of methicillin-resistance *S. aureus* M7 strain (Figure 5B,C), which is one of the most pathogenic bacterium resistant to multiple drugs, having acquired resistance to a variety of them.

Antibacterial studies were also performed against a no foodborne Gram-negative pathogen *Pseudomonas protegens* N, a widespread plant-protecting bacterium isolated from water samples of an irrigation well located in the region of Djebira in Bejaia, northern Algeria [34,35]. The obtained results clearly demonstrated that all the plant extracts under investigation were ineffective to inhibit the growth of the soil microorganism, confirming that the *L. europeaus*-antibacterial proteins appeared to be less potent both versus pathogenic and not pathogenic Gram-negative bacteria. In accordance with the reported findings concerning the screening of antimicrobial potentiality and taking into account the sensitivity of the tested bacteria, extract 2 and *S. aureus* MRSA M7 were chosen to perform the further analyses.

### 2.5. Spectroscopic Analysis

Many of the colors associated with higher plants are due to the presence of pigment molecules, such as chlorophylls and the carotenoids, which confer them a natural fluorescence. Therefore, the intense color of these pigments makes them ideal candidates for absorption spectroscopy studies, having a unique visible spectrum, which can provide a positive identification [36]. In this context, the pigment content in terms of chlorophyll a, chlorophyll b and carotenoids present in the plant extract 2 was determined by spectrofluorometric analysis, performing their extraction using ethyl acetate as solvent that is considered the best extractant for this class of molecules [37]. As shown in Figure S1, the photosynthetic fluorescence emission spectra obtained from the organic extracts evidenced the presence of three main bands: one of chlorophyll a at 650–684 nm, the second at 642–670 nm, characteristic to chlorophyll b, and the last one at 500–600 nm probably due to carotenoids. The same experiment was performed on the plant extract 2 after dialysis in bags with 10 kDa MWCO (Molecular weight cut-off), revealing that it was completely abolished the fluorescence emission peaks corresponding to the three pigment molecules, which were lost during dialysis (Figure S1). Therefore, it is reasonable to assess that the strong antibacterial activity measured in the extract 2, whose preparation includes dialysis, can be attributed to compounds with a molecular mass higher than 10 kDa.

### 2.6. Partial Purification of the Active Compounds

**Figure 6:**
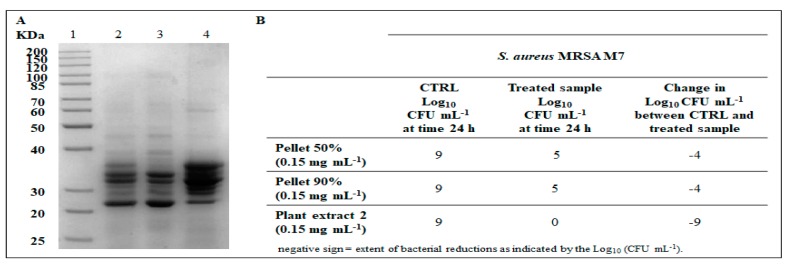
(**A**) SDS-PAGE analysis of protein fractions. Lane 1: molecular weight markers; Lane 2: plant extract 2; Lane 3: protein sample obtained by 50% ammonium sulphate precipitation; Lane 4: protein sample obtained by 90% ammonium sulphate precipitation. Equal amounts of total proteins were loaded for each lane. The gel is representative of three independent experiments on three different protein preparations. (**B**) antibacterial effect of pellet 50%, pellet 90% and plant extract 2 samples against *S. aureus* MRSA M7 reported in terms of change in the Log CFU·mL^−1^ of viable colonies observed between control and treated bacteria at 24 h. Data are representative of three independent experiments on three different protein preparations.

With the aim to gain insight into the protein component/s responsible for the antibacterial activity of the extract 2, a partially purification procedure was performed by a combination of ammonium sulphate fractionation and gel filtration chromatography. In the first step, precipitation experiments were conducted subjecting the extract 2 to precipitation using two sequential salt saturation levels (50% and 90%). The pellets resulting from the two precipitation steps were dissolved in 50 mM sodium acetate buffer pH 5.0, extensively dialyzed to remove the ammonium sulphate, tested for antibacterial activity and analyzed by SDS-PAGE (Figure 6A).

In vitro antibacterial assessment of the two precipitates (named pellet 50% and pellet 90%) was carried out at the MIC value (0.15 mg·mL^−1^) determined with the total extract 2 against *S. aureus* MRSA (Figure 5A) and the results were reported in terms of the change in the Log CFU·mL^−1^ of viable colonies. The bactericidal activity was defined as being equal to 3 Log CFU·mL^−1^ or greater reduction in the viable colony count relative to the initial inoculum [38]. As shown in Figure 6B, a rapid reduction in the log of the viable cells counted (−4 Log CFU·mL^−1^), was detected with both samples. This acknowledged the fact that the bactericidal activity measured for the extract 2 resulted from the contribution of different protein components. However, given that the total protein yield in the 90% pellet was 5-fold lower than that obtained in 50% sample and taking into account the large amount of the starting material required to allow more detailed investigations, we firstly decided to proceed to the purification of 50% pellet. An important aspect to underline is the complete recovery of the proteins responsible of the antibacterial activity in the plant crude extract after precipitation by ammonium sulphate.

**Figure 7:**
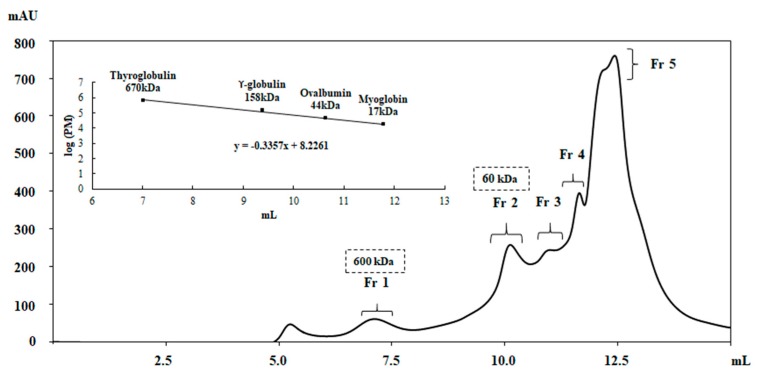
Elution profile of pellet 50% sample obtained by gel filtration chromatography performed on YARRA™ SEC-4000 column in 50 mM sodium acetate buffer pH 5.0 containing 50 mM NaCl. Insert: Calibration curve of the gel filtration YARRA™ SEC-4000 column using protein standards of known molecular masses. The collected fractions are indicated.

**Figure 8:**
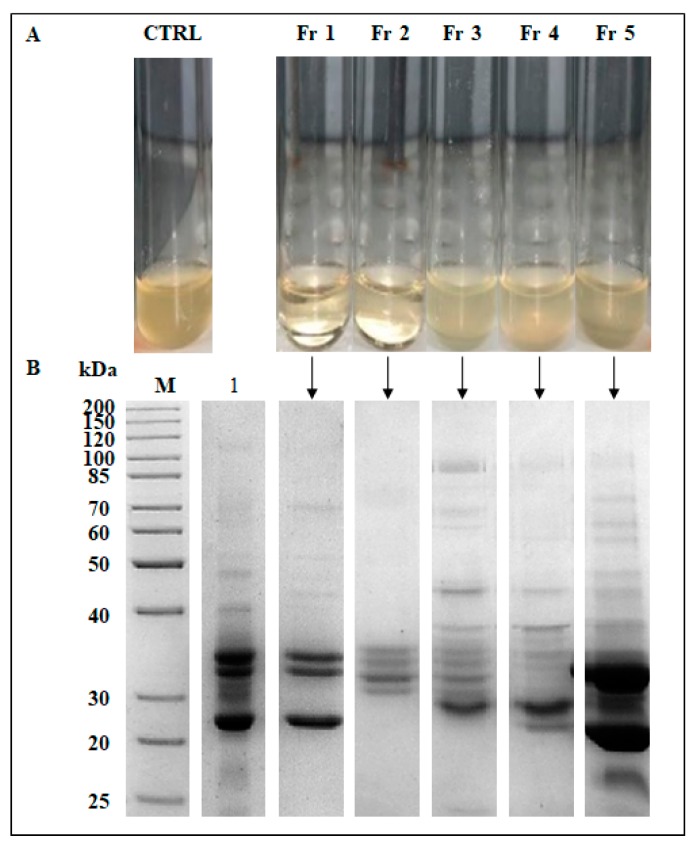
(**A**) Antimicrobial screening assay of gel filtration fractions against *S. aureus* MRSA M7. **CTRL**: *S. aureus* MRSA M7 control; **Fr 1, Fr 2, Fr 3, Fr 4 and Fr 5**: fractions obtained after gel filtration chromatography of the pellet 50% sample. (**B**) SDS-PAGE analysis of the protein fractions. M: molecular weight markers; Lane 1: pellet 50% sample. Equal amounts of total proteins were loaded for each Lane.

Additional purification step was conducted through the gel filtration chromatography on an SEC-4000 column. The elution profile (Figure 7) obtained from 50% pellet, showed five main protein fractions, which were assayed for the antibacterial activity against the *S aureus* MRSA (Figure 8A). A strong killing activity was exhibited by both protein fractions Fr 1 and Fr 2, with MIC values of 0.01 mg·mL^−1^ and 0.04 mg·mL^−1^, respectively, which coincided with the MBCs. In contrast, no activity was observed with the remaining protein fractions Fr 3, Fr 4, and Fr 5. Based on the calibration curve of the gel filtration column, Fr 1 and Fr 2 displayed a molecular mass of approximately 600 kDa and 60 kDa, respectively.

On the other hand, the SDS-PAGE analysis of all the gel filtration fractions revealed not only an enrichment of the active compounds (Fr 1 and Fr 2) (Figure 8B) but also a possible oligomeric nature of the antibacterial proteins considering the molecular mass determined under native conditions (Figure 7).

However, it cannot be excluded that more than one active protein compound could cooperate and contribute to the intrinsic antibacterial activity of the *L. europaeus* plant fruits.

## 3. Materials and Methods

### 3.1. Collection of Plant Material

Shoots of berries of European mistletoe parasitic plant *Loranthus europaeus* were collected between December 2017 and March 2018 from infected oaks (*Quercus pubescens*) in the forest of Valle del Torrente, Savucchia, Carpanzano (Cs), Calabria, South Italy, Italy (39.14453° N–16.31390° E) at about 3–4 meters from the ground level. All the picked samples were yellow-berried leafless aerial shoots. The berries were rounded with a diameter of 0.5–1 cm and were stored at −80 °C until protein extraction. The yellow berries were gently collected by the Prof. Angelo Genovese of University of Naples “Federico II”.

### 3.2. Preparation of Crude Extracts

Total protein extraction from yellow berries was carried out using three protocols. *L. europaeus* frozen berries were pitted and finely powdered in liquid nitrogen using a mortar and pestle and the pulverized mixtures were used for all the extraction protocols. Specifically, protein extracts were obtained by adding to the powdered materials a defined volume of each extraction buffer: 100 mM Tris-HCl pH 8.0, 1 mM DTT, 1 mM PMSF (extract 1); 100 mM Sodium Acetate pH 5.0, 1 mM DTT, 1 mM PMSF (extract 2); 100 mM Tris-HCl pH 8.0, 10 mM EDTA, 10 mM DTT, 5% SDS (extract 3). The extracts 1 and 2 were shaken on a rotatory shaker for 16 h at 4 °C and then centrifuged at 16,000× *g* for 40 min at 4 °C. The extract 3 was shaken on a rotatory shaker for 3 h at 4 °C and then centrifuged at the same conditions described above. All the resulting supernatants were collected and extensively dialyzed in bags with 10 kDa MWCO (Molecular weight cut-off) at 4 °C against 50 mM sodium acetate pH 5.0 for extract 2 and 50 mM Tris-HCl pH 8.0 for extract 1 and 3. The extracted proteins were stored at 4 °C in 5% glycerol until use. The protein concentration was determined according to Bradford’s [39] method using bovine serum albumin as standard.

### 3.3. Antifungal Activity Assays

The antifungal activity of the three extracts was evaluated against four phytophatogenic fungi (*Aspergillus niger*, *Botrytis cinerea*, *Penicillium spp.,* and *Alternaria spp.*) as described in Agrillo et al. [34]. Before the antifungal testing, the protein extracts 1, 2 and 3 were sterilized by filtration through 0.22 μm sterile filters (Millex GV). Tests were performed pouring the extracts (300 µL) in wells (0.5 cm in diameter) aseptically punched on the PCA plates, previously scraped with fungi spores (2 × 10^4^ conidia/mL) and by incubating the plates for 48 h at 28 °C. The antifungal activity was evaluated measuring the diameter of the inhibition zone on PCA plates.

### 3.4. Bacterial Culture and Inoculum Preparation

Methicillin-resistant *Staphylococcus aureus* (MRSA, M7), *Staphylococcus aureus* (MSSA, SA4), *Listeria monocytogenes* (92), no pathogenic *E. coli* (O157) and *Salmonella* Typhimurium isolated from different foods, were used in the microbiological assays. Bacterial cultures were stored at −80 °C. Before the experiments, the frozen stocks of each strain were plated on selective agar and incubated at 37 °C for 16 h to obtain single colonies. Working cultures were produced daily by transferring a loopful of culture to Tryptic Soy Broth (TSB, Biotec, Grosseto, Italy) and incubating for 16 h at 37 °C. To obtain the bacterial suspension, the density of the cell was assessed spectrophotometrically (OD_600_) and the solution was adjusted to 0.1. Enumeration of the inoculum was completed by diluting to approximately 3.0 Log CFU·mL^−1^ and spread-plating 100 μL on selective plate agar. Plates were aerobically incubated at 37 °C for 48 h.

### 3.5. Antibacterial Activity Assay of Plant Fruit Extracts

Minimum inhibitory concentration (MIC) and minimum bactericidal concentration (MBC) were carried out on berry extract 1 and 2 (after dialysis against sodium acetate at 50 mM) according to Clinical and Laboratory Standards Institute (CLSI, 2015), with some modifications [40]. The stock solution (0.50 mg·mL^−1^) of the two berry extracts was diluted at different concentrations (from 0.41 to 0.01 mg·mL^−1^.) in Tryptic Soy Broth (TSB; Biotec, Grosseto, Italy) to a total volume of 1 mL for each tube. 10 μL of each strain, 1.0 × 10^4^ CFU·mL^−1^, was inoculated. At the same time, equal volumes of sterile Tryptic Soy broth were inoculated as a control. The tubes were incubated for 20 ± 2 h at 37 °C and thereafter observed for turbidity. MIC is defined as the lowest concentration of the extract at which no bacterial growth was detected. MBC is defined as the lowest concentration of peptide at which more than 99.9% of the bacterial cells are killed. To determine the MBC, 100 μL of the bacterial cell suspension was taken based on the MICs, cultivated on agar plate and then incubated for 24–48 h at 37 °C. At least six technical replicates were included for each group, and all experiments were performed in triplicate.

### 3.6. Antibacterial Activity Assay of Partially Purified Samples

The antimicrobial efficacy of the partially purified samples (pellets 50% and 90% and gel filtration fractions) was determined according to Palmieri et al. [41]. The pellets were tested at concentration of 0.15 mg·mL^−1^ *versus Staphylococcus aureus* (MRSA, M7). Gel filtration fractions were assayed at concentrations ranging from 0.01 mg·mL^−1^to 0.04 mg·mL^−1^. Under all the experimental conditions explored, the plate counting method was used to estimate the activities.

### 3.7. Partial Purification of the Active Components

Precipitation by ammonium sulphate, a method of protein purification, was performed on total protein extracts 2 followed by gel filtration chromatography to isolate the antibacterial compounds. Powdered ammonium sulphate was added in small portions under constant stirring at 4 °C to 50% and 90% saturation levels. Specifically, the precipitate by ammonium sulphate at 50% saturation was collected by centrifugation at 15,000× *g* for 30 at 4 °C, dissolved in 50 mM sodium acetate pH 5.0 and extensively dialyzed in bags with 10 kDa MWCO (Molecular weight cut-off) against the same buffer to completely remove the salt. The supernatant resulting from the precipitation by 50% was precipitated at 90% (NH4)_2_SO_4_ saturation at 4 °C. After centrifugation at 15,000× *g* for 30 at 4 °C, the resulting pellet was re-suspended in 50 mM sodium acetate pH 5.0 and extensively dialyzed against the same buffer in bags with 10 kDa MWCO (Molecular weight cut-off). All fractions obtained following ammonium sulphate precipitations were tested for antibacterial activity as described above. The sample obtained from salt precipitation which resulted active in the antibacterial tests were loaded on a gel filtration column Yarra 3µm Sec-4000 column (Pharmacia Biotech, Milan, Italy) connected to an HPLC system (Shimadzu, Milan, Italy) and pre-equilibrated in 50 mM sodium acetate containing 50 mM NaCl, pH 5.0. The samples were eluted at a flow rate of 0.5 mL·min^−1^. Fractions were pooled, concentrated and tested for the antibacterial activity as previously described.

### 3.8. SDS-PAGE Analysis

In order to monitor purity, electrophoretic analyses were performed on 10% polyacrylamide gel under denaturing conditions (SDS-PAGE) according to the procedure described by Laemmli [42]. Standard proteins (Page rule Unstained ladder) were purchased from Thermo Scientific (Massachusetts, USA). 

### 3.9. Spectroscopic Analyses

The fluorescence of plant fruit pigments was determined using a Jasco FP-8200 spectrofluorometer. The extraction of the main pigments were performed on the plant extract 2 before and after the dialysis using ethyl acetate as solvent. The extract obtained was centrifuged at 16,000× *g* for about 10 minutes. The supernatant was collected and the fluorescence emission spectra were collected at 25 °C in a 1 cm path length quartz cuvette using excitation and emission slit widths of 2.5 nm. The samples were excited at different λexc and the emission ranges used were: 500–800 nm (480-nm λexc), 600–800 nm (425-nm λexc), and 600–800 nm (470-nm λexc).

### 3.10. Statistical Analysis

All experiments were performed at least five times. Statistical analysis was carried out using the software GraphPad Prism^®^, version 6 (GraphPad, San Diego, California, USA). Statistical analysis of microbiological data was performed by using Student’s t-test (*p* < 0.05) and the results were presented as mean ±standard deviation (s.d.).

## 4. Conclusions

Food spoilage is often caused by the growth of many pathogenic bacterial strains. As a rule, prevention of foodstuff is mainly based on the application of chemical preservatives, whose adverse effects on human health have increased the demand for finding effective, healthy safer and natural compounds [43]. In this context, the plants and their products are gaining a wide interest in the food industry for their potential as decontaminating agents, as they are Generally Recognized as Safe (GRAS) [44].

In the current in vitro study, antimicrobial activity of different protein extracts from *L. europeaus* yellow berries was examined against fungal phytopathogens, Gram-positive and Gram-negative bacteria. Among the investigated protein extracts, the findings clearly revealed that a protein sample containing bioactive constituents, exhibited a remarkable inhibitory activity against two Gram-positive bacteria, *L. monocytogenes* and the methicillin-resistant *S. aureus* M7 strain, being the latter the most susceptible. In addition, a partially purification of this plant fruit extract allowed to identify at least two protein compounds responsible for the efficient bactericidal activity against *S. aureus* M7. Moreover, to the best of our knowledge, this is the first study aimed at the identification of proteins present in the fruits of *L. europeaus* showing bactericidal activity. This work represents a pilot study and confirms that the traditional medicinal plants can be considered an important and rich source of naturally occurring products against common pathogenic microorganisms, thus representing an economic and safe alternative to treat human diseases.

## Supplementary Materials

The following is available online at https://www.mdpi.com/2079-6382/9/2/47/s1, Figure S1: Fluorescence emission spectra of pigments extract from plant extract 2.
