# Supplementary material for: The Bactericidal Activity of Protein Extracts from Loranthus europaeus Berries: A Natural Resource of Bioactive Compounds
Source: Antibiotics (Basel). 2020 Jan 28;9(2):47. doi: 10.3390/antibiotics9020047 (PMC7168301; doi:10.3390/antibiotics9020047)
Supplement: Supplementary file 1 [file antibiotics-09-00047-s001.pdf]

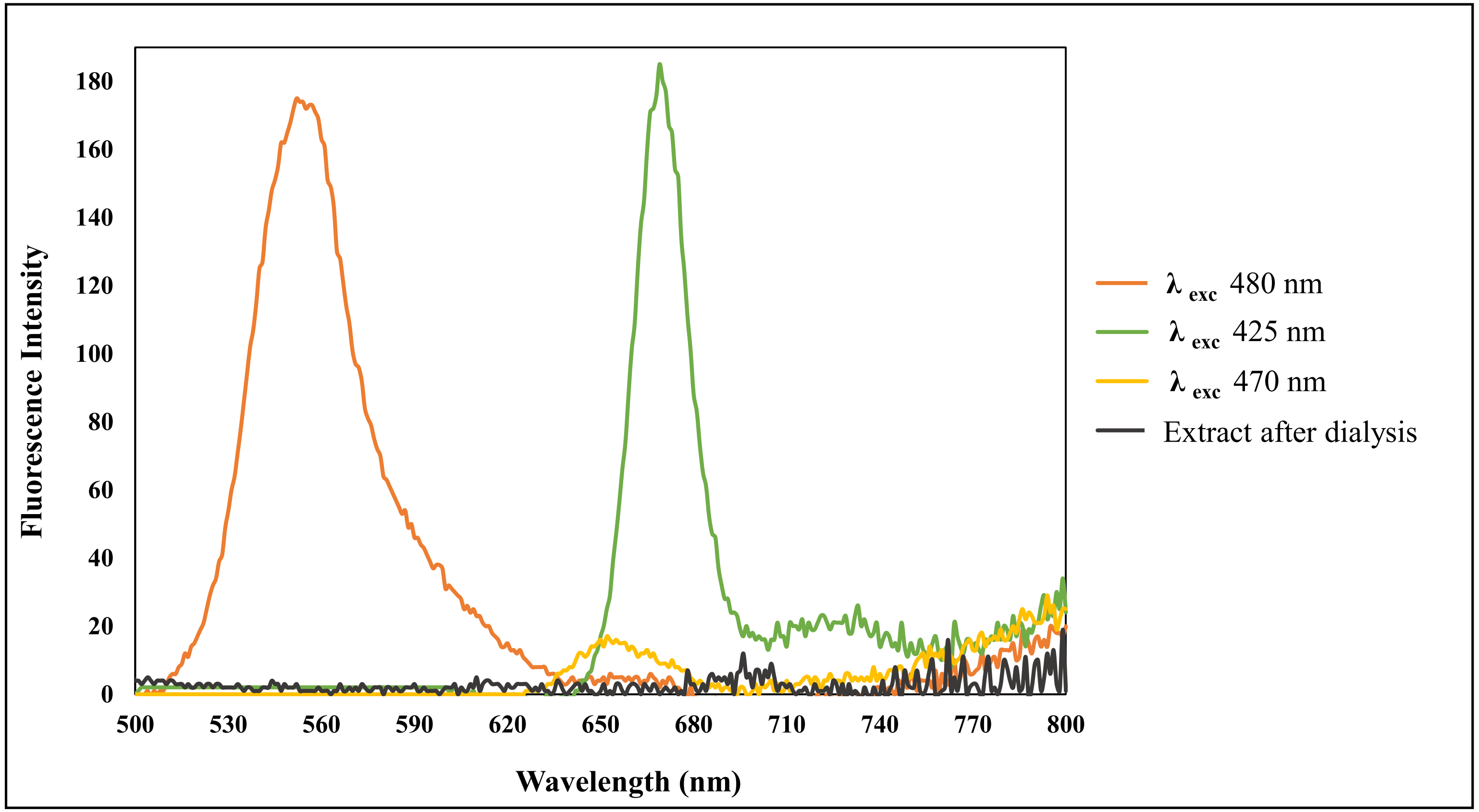

Figure S1. Fluorescence emission spectra of pigments extracted from protein fraction obtained by method 2, before and after dialysis of the sample, at different excitation wavelengths.
